# 

**Published:** 1876-03

**Authors:** 


### Meeting of the American Medical Association.

The Twenty-seventh Annual Session of the American Medical Association will be held in the city of Philadelphia, Pa., on Tuesday, June 6, 1876, at 11 A. M.

" The delegates shall receive their appointment from permanently organized State Medical Societies, and such County and District Medical Societies as are recognized by representation in their respective State Societies, and from the Medical Department of the Army and Navy of the United States."

" Each State, County, and District Medical Society entitled to representation shall have the privilege of sending to the Association one delegate for every ten of its regular resident members, and one for every additional fraction of more than half that number ; Provided, however, that the number of delegates for any particular State, territory, county, city, or town shall not exceed the raito of one in ten of the resident physicians who may have signed the Code of Ethics of the Association."

☞Secretaries of Medical Societies as above designated are earnestly requested to forward at once, lists of their delegates to the Secretary, Dr. Atkinson, in order that the Committee of Arrangements may be able to form some idea of the number likely to be present.

The following Committees are expected to report :—

On Mechanism of Accommodating the Eye, Dr. D. S. Reynolds, Ky., Chairman. On New Remidies, Dr. Austin Flint, Jr., N. Y. Chairman. On the Medical and Surgical Uses of the Aspirator, Dr. E. S. Gaillard, Ky., Chairman. On Influence of Climate on Pulmonary Diseases in Minnesota, Dr. Franklin Staples, Minn., Chairman. On the same in Florida, Dr. E. T. Sabal, Fla., Chairman. On Proper Legislation to Prevent the Spread of Syphilis, Dr. Samuel D. Gross, Pa., Chairman. On Prize Essays, Dr. Samuel D. Gross, Pa., Chairman. On Neorolocy, Dr. S. C. Chew, Md,, Chairman. On Rank of Medical Corps of the Army, Dr. H. A. Johnson, Ill., Chairman.

### Books and Pamphlets Received.

A Manual of General Pathology, for the Use of Students and Practitioners of Medicine. By Ernst Wagner, M. D., Leipzig. Translated from the Sixth German Edition by John Van Dengn, A. M., M. D., and E. C. Seguin, M. D. New York: Wm. Wood & Co. 1876. Buffalo: H, H. Otis.

Medical Thermometry and Human Temperature. By E. Seguin, M. D. New York: Wm. Wood & Co. Buffalo: H. H. Otis.

Transactions of the Thirtieth Annual meeting of the Ohio State Medical Society, held at Put-in-bay, June, 1875.

Filth-Diseases and their Prevention. By John Simon, M. D., F. R. C. S Printed under the State Board of Health of Massachusetts. Boston: James Campbell. 1876.

Insanity in its Medico-Legal Relations. By A. C. Cowperthwait, A. M., M. D. Philadelphia: J. M. Stoddart & Co. 1876.

### Contents of March Number.

### ORIGINAL.

THE BUFFALO

**Medical and Surgical Journal**

PUBLISHED MONTHLY,

Containing Original Articles, Reports of Medical Societies and Hospitals, Editorial, Reviews, Correspondence, Army News, etc., etc.; including the usual variety of Medical Periodical Publications. Specimen copies sent on application.

Address Buffalo Medical & Surgical Journal, Buffalo, N. Y.

TERMS OF SUBSCRIPTION, $3.00 PER YEAR, IN ADVANCE.

